# Mobile element insertions are frequent in oesophageal adenocarcinomas and can mislead paired-end sequencing analysis

**DOI:** 10.1186/s12864-015-1685-z

**Published:** 2015-07-10

**Authors:** Anna L. Paterson, Jamie M.J. Weaver, Matthew D. Eldridge, Simon Tavaré, Rebecca C. Fitzgerald, Paul A.W. Edwards, the OCCAMs Consortium

**Affiliations:** Department of Pathology, University of Cambridge, Hutchison-MRC Research Centre, Cambridge, UK; MRC Cancer Unit, Hutchison-MRC Research Centre, University of Cambridge, Cambridge, UK; Department of Pathology, Addenbrookes Hospital, Cambridge, UK; Cancer Research UK Cambridge Institute, University of Cambridge, Cambridge, UK

**Keywords:** Mobile elements, Cancer, Oesophageal adenocarcinoma, Paired-end DNA sequencing

## Abstract

**Background:**

Mobile elements are active in the human genome, both in the germline and cancers, where they can mutate driver genes.

**Results:**

While analysing whole genome paired-end sequencing of oesophageal adenocarcinomas to find genomic rearrangements, we identified three ways in which new mobile element insertions appear in the data, resembling translocation or insertion junctions: inserts where unique sequence has been transduced by an L1 (Long interspersed element 1) mobile element; novel inserts that are confidently, but often incorrectly, mapped by alignment software to L1s or polyA tracts in the reference sequence; and a combination of these two ways, where different sequences within one insert are mapped to different loci. We identified nine unique sequences that were transduced by neighbouring L1s, both L1s in the reference genome and L1s not present in the reference. Many of the resulting inserts were small fragments that include little or no recognisable mobile element sequence. We found 6 loci in the reference genome to which sequence reads from inserts were frequently mapped, probably erroneously, by alignment software: these were either L1 sequence or particularly long polyA runs. Inserts identified from such apparent rearrangement junctions averaged 16 inserts/tumour, range 0–153 insertions in 43 tumours. However, many inserts would not be detected by mapping the sequences to the reference genome, because they do not include sufficient mappable sequence. To estimate total somatic inserts we searched for polyA sequences that were not present in the matched normal or other normals from the same tumour batch, and were not associated with known polymorphisms. Samples of these candidate inserts were verified by sequencing across them or manual inspection of surrounding reads: at least 85 % were somatic and resembled L1-mediated events, most including L1Hs sequence. Approximately 100 such inserts were detected per tumour on average (range zero to approximately 700).

**Conclusions:**

Somatic mobile elements insertions are abundant in these tumours, with over 75 % of cases having a number of novel inserts detected. The inserts create a variety of problems for the interpretation of paired-end sequencing data.

**Electronic supplementary material:**

The online version of this article (doi:10.1186/s12864-015-1685-z) contains supplementary material, which is available to authorized users.

## Background

**Fig. 1:**
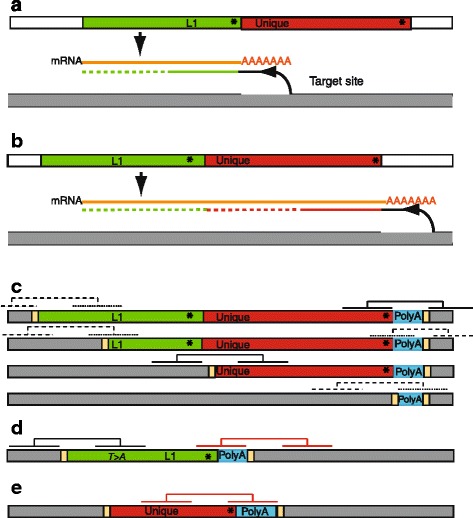
Inserts produced by L1 activity and how they are treated by paired-end sequencing. **a, b** Generation of inserts showing truncation and transduction (not to scale). mRNA (orange) is transcribed from an L1 in the germline (or from a newly inserted L1, if complete). Target site is nicked, and the end at the nick used to prime reverse transcription of the mRNA to cDNA (green and red), which is often incomplete (dotted line). cDNA is subsequently integrated, flanked by a short duplication of the target site. Some inserts have 5’ inversions, perhaps due to additional priming in the opposite direction [19]. **a**, simple L1 insert; **b**, Transduction of 3’ unique sequence. Transcription of an L1 sometimes reads through the L1 polyA addition site (asterisk) into 3’ unique sequence (red) until a polyA addition site (asterisk) is encountered. The resulting cDNA and insert includes a variable amount of the unique sequence and upstream L1 sequence. **c** Examples of inserts with transduced unique sequence, and resulting paired-end sequence reads. Reverse transcription of the mobile element RNA is often incomplete, resulting in 5’ -truncated inserts. These may or may not have any L1 sequence, and the most-truncated inserts may contain little more than polyA. Examples of possible read pairs are shown in black solid lines if the aligner can map (align) them uniquely to the reference genome; these will usually appear to be translocation junctions (Fig. 2). Many read pairs will not align (dashed lines) either because one read falls in a repeat, or the sequence is not present in the reference (fine dots). Yellow boxes are target site duplications. **d** Example of an insert of an L1 that does not transduce 3’ sequence but nevertheless may be mapped as a translocation junction. The parent L1 may have a unique sequence difference, e.g. a single base pair deviation (*T > A*) from the consensus, that identifies reads uniquely and maps them to its parent L1. Other reads (red) are aligned to an L1 (or Alu) in the reference sequence that has a polyA tail, e.g. the ‘element’ on chromosome 15 in Table 1. In some cases the alignment may be generated by the polyA alone. Such inserts are not in general from the element the read is mapped to. **e** Apparent junction that is not even a junction. Occasionally a read pair within an insert may be aligned to two different loci, appearing to report a rearrangement junction. For example, one read may map to a transduced sequence, while its pair contains polyA and is mapped to one of the polyA runs in the reference genome

Mobile elements are elements in the genome that can move, either by excision and re-insertion of the DNA itself or by insertion of copies made by reverse transcription of an mRNA intermediate [1–3]. The most active in humans are the non-retrovirus-like retrotransposons, the LINE and SINE elements, including respectively L1s and Alus. Intact, active L1s are transcribed into mRNA, which has two open reading frames. The encoded enzyme activities include an endonuclease and reverse transcriptase. The endonuclease cuts the genome at a consensus target site, exposing an end that is used to prime reverse transcription of the L1 mRNA, and the resulting cDNA is integrated (Fig. 1A). Alus and SVAs do not encode the necessary enzymes and can only be copied when enzymes are provided by an intact L1 [4]. Other mRNAs can also be inserted by L1 enzymes to give novel processed pseudogenes [5]. In humans, a number of L1 elements are intact and active, and create novel insertions: in the germline—causing polymorphism and constitutional genetic disease [1–3]; in cancers [6–14]; and probably also in non-cancerous somatic tissues (e.g. [4, 15]). Although there are limited data so far, there is an expectation that mobile element insertions will contribute to mutation of driver genes in cancers.

Two important features of L1 retrotransposition are truncation and transduction (Fig. 1) [3]. The inserted copies are often 5’ truncated; and inserts often include sequence from downstream of the L1 element proper, because transcription may extend beyond the end of the L1 element, to a downstream polyA addition signal (Fig. 1B) [1, 13, 16, 17]. Thus the insert usually consists of polyA, with a variable amount of upstream sequence, which may or may not include part of the L1 sequence itself (Fig. 1C) [18]. The inserts may also be partly inverted, possibly as a result of priming reverse transcription from both directions [19].

Paired-end sequencing is currently the method of choice for detecting genome rearrangements in cancer or germline DNA [20, 21]. Typically, sequence reads of 100bp are taken from both ends of genomic DNA fragments of 200-500bp. The reads are then aligned to the reference genome, allowing for some variation. Rearrangements are detected mainly by looking for ‘discordant read pairs’, i.e. read pairs where the two reads do not map to the same place in the genome at the expected separation, e.g. to different chromosomes. Reads that cross rearrangement junctions, ‘split reads’, may also be searched for. Thus central to the analysis of the sequencing data is correct interpretation of the alignment of discordant read pairs, or ‘split reads’, to the genome.

The present study is part of a programme to sequence the genomes of 500 oesophageal adenocarcinomas [22] within the framework of the International Cancer Genome Consortium.

While analysing genomic rearrangements of these tumours from paired-end sequencing data, we, as others working with other tumour types [12, 13], identified a puzzling class of genome rearrangement. Typically, several tumours appeared to have chromosome translocation breakpoints at the same place, to within about a kilobase, apparently translocated to, or inserted into, many places in the genome, in a tumour-specific way, and often with multiple translocations in the same tumour.

We hypothesized that these ‘rearrangements’ were insertions of mobile elements, detected in paired-end sequencing because part of the sequence inserted was unique in the genome.

## Results

### Identification of candidate mobile element transduction events

**Fig. 2:**
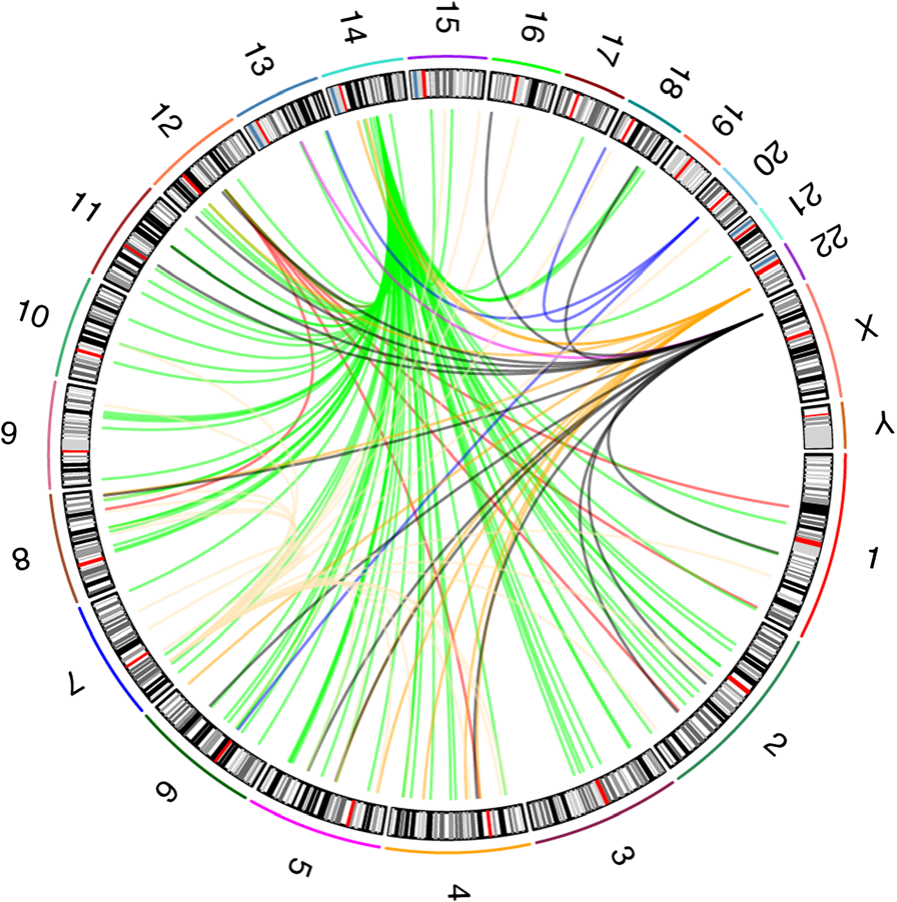
Mobile element inserts mimic multiple translocations. Circos plot of mobile element inserts detected by discordant read pairs in tumour 7409, which had the highest number detected. The genome is displayed as a circle, chromosomes 1 to Y, with curved lines representing the apparent rearrangements detected. For example, the many apparent junctions between chromosome 14 and other chromosomes (green), represent copies of a chromosome 14 sequence that have been transduced and inserted all over the genome

As others have reported [12], among the rearrangement junctions in our paired-end sequencing were groups of apparent translocations that had highly recurrent breakpoints, ‘translocated’ to many unique sites (Fig. 2). For example, 17/22 tumours (in our discovery set Batch P) had a total of 61 ‘translocation breakpoints’ within a 1.4kb region at 29.065 Mb (reference genome GrCH37/hg19) on chromosome 22, within an intron of the *TTC28* gene, up to seven in the same tumour, and the joined ‘breakpoints’ were all different. Similarly, four tumours had a total of 46 junctions at 59.220 Mb on chromosome 14 and seven tumours had a total of 29 at 11.732 Mb on chromosome X (Table 1, Additional file 1). These could be distinguished from typical artefactual ‘translocations’ resulting from misalignment of reads to repeat sequences, because usually both ‘breakpoints’ of such events are joined to multiple partner breakpoints. They also did not resemble true recurrent translocations because the breakpoints were clustered into too small a region, generally 1kb or less. For many—typically a quarter to a half—of the junctions, paired-end sequencing detected a neighbouring junction, suggesting that the ‘translocation’ was reciprocal, perhaps with a small duplication at the breakpoint [23], or was in fact an insertion. For example, 26 of the 61 chromosome 22 ‘translocations’ had two junctions (Additional file 1).

**Table 1:** ‘Elements’ in the reference genome that mobile element inserts align to

ID	Chromo-some	Transduced sequence start	Transduced sequence end	L1, Alu etc.	Start	End	+/−	Gene	Total Inserts	Tumours	Method	Max Transduced (bp)	Hot L1 list	Tubio list
Reference L1s that transduce unique sequence														
Chr 4	chr4	137213864	in L1	L1HS	137214650	137220701	-	none	3	2	L1	786	14	No
Chr 8	chr8	135082457	135082642	L1HS	135082987	135089016	-	none	6	3	L1	530	3	4
Chr 12_A	chr12	3606945	in L1	L1HS	3608362	3614394	-	PRMT8	5	3	L1	1417	35	6
Chr 20	chr20	23412922	23413624	L1HS	23406746	23412777	+	none	11	5	Cl	847	Not listed	8
Chr 22	chr22	29065365	29066424	L1HS	29059272	29065303	+	TTC28	129	36	Cl, L1	1121	7	137
Chr X_A	chrX	11731785	11732702	L1HS	11725369	11731399	+	none	80	19	Cl, L1	1303	19	7
Chr X_B	chrX	11952984	in L1	L1HS	11953208	11959433	-	none	23	6	L1	224	1	20
*Non -* reference L1s that transduce unique sequence														
Chr 3	chr3	*in L1*	*123595443*	Polymorphic L1HS	*123590726*	*123590724*	+	MYLK	29	7	Cl	>4000	N/A	40
Chr 14	chr14	59220410	59221078	Polymorphic L1HS	*59220404*	*59220404*	+	none	179	11	Cl		N/A	40
Reference genome elements that inserts align to														
Chr 6	chr6			L1HS	24811907	24817934	-	FAM65B	3	3	L1	Nil	2	N/A
Chr 7	chr7			L1HS plus 4bp and polyA	30478859	30484914	+	NOD1	84	9	L1	Nil	18	N/A
Chr8		see	footnote	L1HS										
Chr 10	chr10			L1HS with polyA	111572121	111578215	-	none	6	6	L1	Nil	9	N/A
Chr 12_B	chr12			AluSx1 with polyA	66451373	66451739	-	none	98	21	Cl	Nil	N/A	N/A
Chr 15	chr15			AluYa5 with polyA	77910868	77911236	-	LINGO1	19	17	Cl	Nil	N/A	N/A

Positions refer to reference genome GRCh37/hg19. The mobile ‘elements’ are identified by chromosome. At least two inserts were verified by PCR for each ‘element’ except the Chr 6 element (only one insert verified) and the Chr 3 element (not verified but described by Tubio *et al.* [13]). The Chr 3 and Chr 14 elements are polymorphic L1s that are not in the reference genome but were shown to transduce sequence by Tubio *et al.* [13]; their insertion point in the reference genome is given in italics. The polyA addition site for the Chr 14 element is at the end of a 36bp fragment of an L2a element. The Chr8 element showed both transduction and mapping to the native L1 insert—of the two inserts verified, one had 3’ unique sequence transduced and one was pure L1 3’ terminus

Transduced sequence, maximum extent of unique sequence observed in inserts verified by PCR (Additional file 2), except for Chr 3 element where read map position is given. +/−, strand bearing polyA, which is same as orientation of L1 or Alu if present. Tumours, number of tumours with inserts. Method, method of identification: Cl, cluster of ‘translocation’ breakpoints from discordant reads; L1, cluster of breakpoints 3’ to a known active L1 (not exhaustive). Max transduced, maximum unique sequence transduced in cloned insert. Hot L1 list, rank in list of active L1s of Brouha *et al.* [24]. Tubio list, whether the elements that transduce 3’ sequence were listed by Tubio *et al.* [13] and how many inserts were reported; N/A, not applicable as were not transduction events

In the 22 tumours of Batch P, seven distinct loci had at least five of these translocation-like junctions, spread over more than one tumour, each breakpoint within 1kb of the next (marked ‘Cl’ in Table 1; Additional file 1), which were verified by PCR as described below.

We hypothesized that these were insertions of L1 mobile elements, and to explore this idea we searched both Batch P and the 21 Batch B1 tumours for rearrangement junctions that mapped to, or close to, the 3’ ends of the 90 L1s in the reference genome that are thought to be capable of mobilisation (Table four of ref. [24]). This identified nine junction clusters, two of which were among the seven originally identified (Table 1; Additional file 1).

The combined list of apparent inserts was compared to known L1, Alu and SVA polymorphisms [13, 25, 26] and only eight (of nearly 700) ‘insert sites’ corresponded to known polymorphisms that we had failed to sample in the normals (Additional file 1).

Inspection of the inserts suggested two ways in which inserts created by mobile elements could be identified as rearrangements by conventional paired-end-read mapping: the first, when the inserts included unique sequence transduced by the mobile element, and the second, when the insert included sequence that could be—correctly or incorrectly—uniquely matched to a mobile element in the reference genome (Fig. 1).

The first way accounted for several ‘clusters’ of inserts that included unique sequence that mapped 3’ to the end of an L1 in the reference genome (Table 1). The chromosome 22 element was typical—inserts included sequence 3’ to an L1 at chr22: 29059272–29065303, extending to at least 29066424.

Two further clusters of inserts of unique sequence—at chromosome 3: 123.59 Mb and chromosome 14: 59.22 Mb (the most abundant in our original set)—resembled transduced sequence but were not adjacent to L1s in the reference genome. They were adjacent to polymorphic L1s absent from the reference genome [26] and known to transduce 3’ flanking sequence [12, 13] (Table 1). The chromosome 14 element, for example, is inserted in the reference sequence at 59220404 in 25 % of the population sampled by Tubio *et al.* [13], in the plus direction. Our inserts all ended in polyA added at map position 59221073–59221078 (Table 1).

The second way that candidate inserts were detected, was when a cluster of breakpoints was mapped to the 3’ terminus of an L1 or Alu in the reference genome with or without a polyA tail. Although these sequences are ‘repeats’, they are sufficiently distinct in the reference genome that aligners (which do not mask repeat sequences) may align reads confidently to these positions because they match a read better than anywhere else (Fig. 1 D; Table 1). For example, 84 rearrangement junctions, spread over nine tumours, were mapped to the 3’ end of an L1 that ends at 30,484,890 bp on chromosome 7 and is followed by a 4 bp gap then 20As.

Similarly, approximately 100 junctions, in 21 tumours, mapped to the longest polyA run in the reference genome, the 90-bp polyA tail of an Alu at chr12:66451373–66451739, while 22 junctions in 18 tumours mapped to the 79 bp polyA tail of an AluYa5 at chr15: 77910868–77911236. The interpretation of these depended on the aligner used. The Novoalign aligner (Batch B1 tumours), which can trim reads more to achieve a match, generally aligned reads to them solely because they contained 70 or more polyA/T, without Alu sequence, but with or without a few bp 3’ to the polyA that may represent common target site sequence. For example, the read TTATTC[74 polyT]GGGAGAGAGATTTTTTTTTT was aligned to the chromosome 15 locus by matching the TTATTC and polyT, then trimming off the remaining base pairs. Of three tested by PCR for each locus (see below; Additional file 2), all were somatic inserts. Two were essentially only polyA while four included an L1Hs 3’ terminus ignored by the aligner.

The aligner BWA (Batch P tumours) aligned reads to these polyA runs by matching polyA plus some recognisable Alu sequence. Their apparent insertion sites were 3’ to reference Alu sequences, and two—at about 49.3603 Mb on chromosome 19 and 141.014 Mb on chromosome 2—were common to two tumours, so these may represent germline polymorphisms of longer polyA tails on these reference Alus that we failed to sample in the matched normals and normal panel.

Thus this second class of apparent rearrangements are where an aligner confidently maps reads to the 3’ end or polyA of a mobile element in the reference genome. These may occasionally represent mobilisations of that reference mobile element, but more commonly will be incorrect mappings of an insert from an unrelated element, or a polymorphism.

A third class of apparent junction, observed only using the Novoalign aligner, appeared to be a special case of such mismappings, which we designated ‘split mappings’. Apparently, reads from within one mobile element insert were mapped to different places in the reference genome (Fig. 1E). For example, we identified a ‘translocation junction’ between chromosome 14:59.2206 Mb, which is sequence transduced by an L1, and the polyA run at chromosome 15:77.9109 Mb (Table 1). Presumably the sequence reads are from an insert of the chromosome 14 L1 that has transduced flanking sequence, and its added polyA tail. We detected ‘junctions’ of this kind that mapped to the chromosome 14 and X_A transduced sequences at one end and either the chromosome 12 or 15 polyA runs at the other. Four other junctions appeared similar, joining the chromosome 14 or 15 loci either to an L1Hs at chr2:88206897–88206997 or sequences found to be transduced by Tubio *et al.* [13] (Additional file 1).

### Verification and structure of inserts

**Fig. 3:**
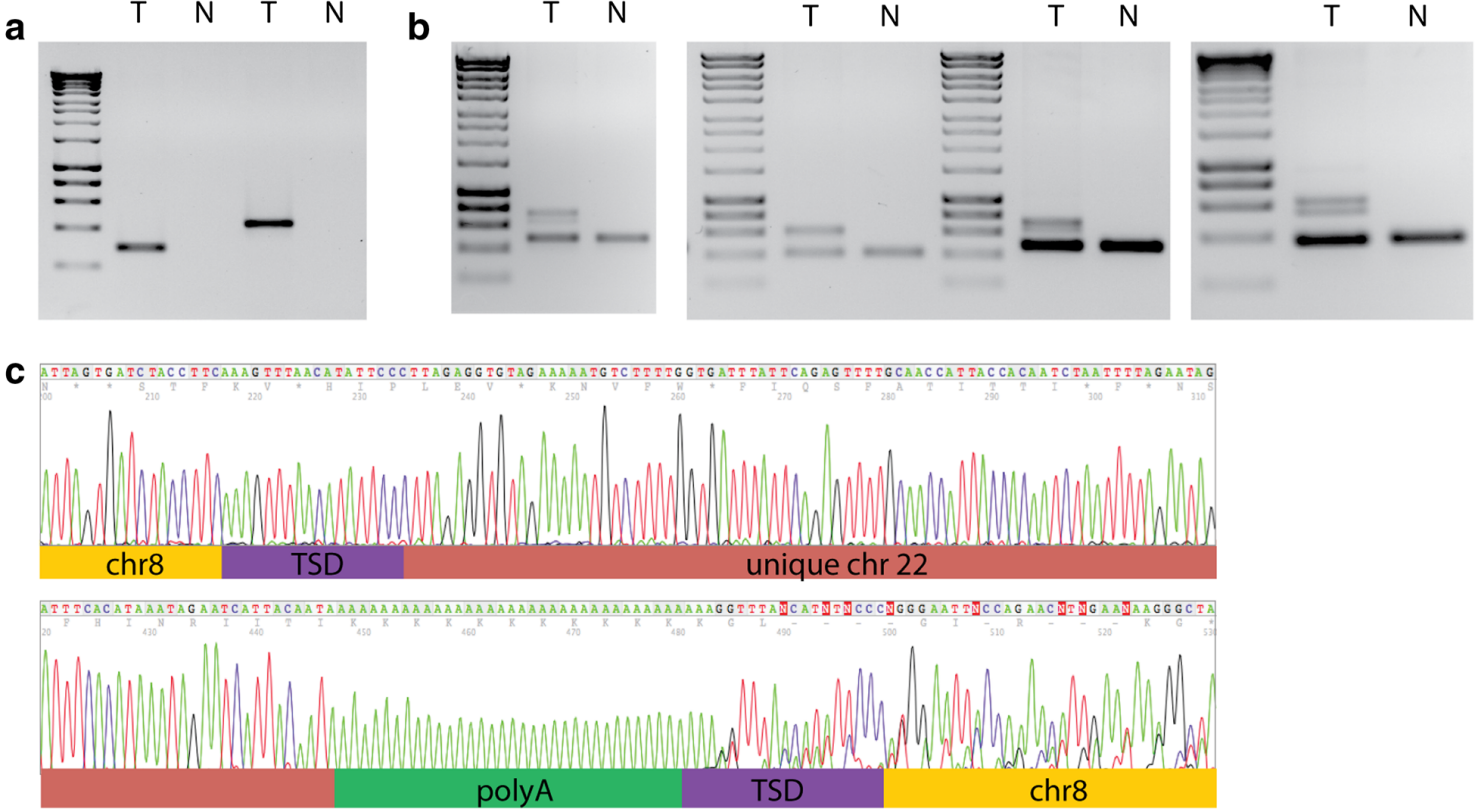
Verification of representative inserts. **a** Flanking junctions of an insert from chromosome 22 into *ENPP2* on chromosome 8 in Tumour 7396. **b** PCR across four representative inserts, of elements denoted Chr X_B, Chr 6, Chr 7 and Chr 14 respectively. Note that in addition to the larger, tumour-specific band in most cases there is an intermediate size band, most clearly in the last example. **c** sequence of insert in **a**, showing target site duplication (TSD), insert of chromosome 22 material and polyA tail

Using PCR, we verified that rearrangement junctions existed and were somatic for at least two inserts from each element in Table 1, except the chr3 element (not attempted) and chr6 (only one attempted). Inserts with read pairs at both ends were preferred (Additional file 1) (this would bias towards truncated inserts, see Fig. 1). For 25/45 inserts we were able to amplify across the insert, using primers designed to the expected flanking sequence (Fig. 3A; Additional file 2). For a further 17, we were able to amplify across at least one junction, giving an overall verification rate of 42/45 tumour inserts (93 %). Failure to amplify some junctions and inserts is not surprising, since some inserts would be too large to amplify and many primers were designed by guessing the other side of an insert, which could have been rearranged or deleted [1]. All inserts were tumour-specific. Amplifying across the inserts gave a product from the normal target site, common to the tumour and matched normal, plus a larger band unique to the tumour. In addition, a weaker intermediate band was often obtained, which on cloning and sequencing gave sequence the same as the normal or insert band and therefore we suspected was a hybrid between the normal and insert bands. Supporting this interpretation, when excised and re-run, the normal and larger insert bands ran as expected, while the intermediate band regenerated the pattern of three bands.

All verified inserts had the expected structure, allowing for known variations. All had a polyA tail, with upstream insert sequence variably truncated (Fig. 3; Additional file 2). Most were flanked by target site duplication, up to 20bp, as expected [10, 13], though some inserts showed zero duplication or even a small deletion (Additional file 2). Variations included partial inversion of the 5’ end of the inserted sequence—about 20 % (36/195) of inserts where both ends were found by discordant read analysis. For example, the insert shown in Fig. 3, of chr22 into chr 8 consisted principally of chr22 sequence, from 29066229–29066424, 3’ to the intact L1 that ends at 29065303, followed by 37bp of polyA (Fig. 3). There was an apparent small inversion at the 5’ end, with the first 17 bp of the insert mapping in the reverse orientation 54 bp upstream to the main chr22 material, consistent with the proposed mechanism for inversion [19]. The insert was flanked by an 18-bp target site duplication.

The inserts we verified and sequenced across ranged from 1bp (plus >50 polyA and target site duplication) to about 1kb excluding the polyA, and the polyA sequences ranged from roughly 25 to over 50 (polymerase slippage means that these figures are approximate). The inserts of transduced 3’ unique sequence included part or all of the transduced unique sequence, with or without part of the upstream L1HS sequence. For example, the insert previously described included no L1HS sequence, while the insert of the chr4 element into chr5 included 69bp of the 3’ end of the (negative-strand) L1HS together with 931bp of unique chromosome 4 material downstream of this end.

The sequences transduced 3’ to intact L1s mostly were bounded by polyA addition sites 0.8-1.3 kb from the L1, though one, from the element at 119.5 Mb on chromosome X, extended only 24bp from the L1. For some the polyA addition sites varied, e.g. for the chromosome 22 element, the L1 of which ends at 29,065,303, there were two cases of polyA addition at 29,065,912, two at 29,066,121, one at 29,066,424, and others. The chr8 element was found both with 3’ unique sequence transduced and without any transduced sequence.

### Estimating abundance of inserts by detecting polyA

Since the total number of L1–mediated insertions is likely to be much greater than detected by discordant read pairs, which only works where reads in the insert can be mapped uniquely to the reference genome, we obtained a rough estimate of total inserts by searching for tumour-specific polyA sequence, hypothesising that most of these would be L1-mediated insertion events.

We searched the 43 tumours of sets P and B1 for reads that contained at least 20 consecutive As or Ts and were paired with sequence reads that were confidently mapped by the BWA aligner. From these we extracted candidate inserts that were supported by at least three read pairs and absent not only from the matched normal but also the 20 or 21 other normals from the same batch of tumours (Additional file 3). We compared these candidate insert sites to known polymorphic L1s and Alus [13, 25, 26], and about 1 % (63 out of approximately 5300) were within 1kb, without considering direction of read. As a control, the analysis was repeated with each B1 tumour interchanged with its matched normal: this detected only an average of 2 (range 0 to 6) candidate normal-specific polyA sequence runs (1/42 of which matched a known polymorphism), suggesting that relatively few of the inserts detected in the tumours were merely bioinformatic artefacts or polymorphisms that we had failed to detect in the matched sample.

We confirmed that the great majority of these candidate insertions resembled tumour-specific L1 events, by manually scrutinizing a random sample of 20 of the least-confident examples, i.e. those supported by only three reads. We viewed all sequence reads within an approximately 600bp window spanning the candidate insert site, using the Integrative Genomics Viewer (IGV) [27] (Additional file 4). All 20 showed additional aberrant reads including additional ‘split’ or ‘soft-clipped’ reads (i.e. reads partially aligned to the reference with a ‘tail’ of mismatched bases) that clearly identified an insertion point. In 18/20 cases these split reads included L1 sequence. In one further case the sequence appeared to be transduced. In only 1/20 cases was the insert clearly germline, while only one further case had a single read in the normal that could possibly have supported a germline insert (Additional file 4).

For around 85 % of an independent unbiased sample of the candidate tumour-specific polyA sequence we were able to verify a retrotransposon insert by PCR across the candidate insert site, and some of the failures might have been inserts that were too large to amplify (Additional file 2). 28 primer pairs were designed, of which 26 pairs successfully amplified a normal band from the matched normal. 22 of these 26 (85 %) amplified a larger band from the tumour. All these insert bands were absent from the matched normal. 11 of these 22 were cloned and sequenced. All showed an insert that included polyA, together with additional sequence (Additional file 2). 10/11 had target site duplication, suggesting they were indeed non-LTR retrotransposon events. 8/11 included the 3’ end of L1HS sequence, for example, the largest was 7416_19, almost 600bp, comprising around 450 bp of L1 sequence, slightly rearranged, with two stretches of polyA separated by a 75bp fragment of chromosome 9, flanked by duplication of TAGAAGCCCAATTTCT. The three without L1 sequence included the smallest, 7436_9, which was ATATAATAATAAT + polyA, flanked both sides by CCCA, which is a duplication of the native chromosome 9 target site. ATATAATAATAAT does not match the end of a consensus L1HS or Alu. Insert 7416_13 comprised 67 bp unique sequence from chromosome 10:120,819,239-120,819,305, plus polyA and duplication of AAGAAATATTTCCC. Insert 7416_10 was about 1kb of unique sequence from chromosome 17 chr17:46707420–46708467, inserted with a target site duplication of 14bp, but with polyA at both ends, A_31_ and A_16_ allowing for the original sequence, in the same orientation. Neither of these two are near reference L1s or known polymorphic L1s [13, 25, 26]. They might represent undocumented polymorphic L1s or re-mobilisation of somatic inserts [13], and, indeed, discordant read pairs show nine rearrangement breakpoints within chr17:46707414–46708480 in tumour 7416, indicating multiple transductions of this sequence.

A further four inserts were also verified, three chosen because they were in genes, *PARK2*, *FOXP2* and *SNTG* (Additional file 2).

Taking 85 % as an estimate of validity, our rough estimate of tumour-specific inserts identified by polyA sequence ranged from five (which may be artefactual background) to about 700, average approximately 100 (Table 2). This is over six times the number of inserts from the elements listed in Table 1 detected as rearrangement junctions. This may be an underestimate because read pairs have to be placed precisely to detect the inserts, and some of the tumours are contaminated enough with normal cells to reduce sensitivity of detection.

**Table 2:** Active elements in individual tumours

Tumour	Element										Elements active	Inserts by discordant reads	Candidate Poly A inserts	TP53 Mutation
	3	4	8	12_A	14	20	22	X_A	X_B	?				
3109	3		1		*26*	1	1				5	32	150	MUT
3111†				1							1	1	96	MUT
3113							2	2		2	3	6	13	MUT
3115							3			1	2	4	33	MUT
3117											0	0	10	MUT
3119											0	0	8	—
3121	4						3			1	3	8	34	MUT
3125											0	0	7	—
3129							6				1	6	129	MUT
3131					1			1			2	2	12	MUT
3133							1			1	2	2	11	—
3135							6			1	2	7	16	MUT
3137							1				1	1	5	MUT
3149					5		3	7			3	15	19	MUT
3302	9						8	3		*21*	4	41	389	MUT
3305							5			2	2	7	16	MUT
3308							6	8			2	14	194	MUT
3311							1	5		1	3	7	95	MUT
3314							4			1	2	5	22	MUT
3317					*14*		4			1	3	19	70	MUT
3320						2	4	2			3	8	97	MUT
3323							3			1	2	4	27	MUT
7394						4	2	7		*14*	4	27	281	MUT
7396	2						*10*	8		*35*	4	55	830	MUT
7398				2	*12*		4	6		*11*	5	35	231	MUT
7401	1				2		2	2	1	3	6	11	138	MUT
7404†				2							1	2	13	MUT
7407					*22*		1	7		3	4	33	196	MUT
7409					*85*	3	*10*	1	*15*	*35*	6	149	416	MUT
7414							1		1	3	3	5	33	MUT
7416					0		4			*11*	2	15	196	MUT
7418							4	3		2	3	9	73	MUT
7420		1	2				6	2	1	1	6	13	100	MUT
7422						1		1	1	4	4	7	62	—
7424	8	2					5			*32*	4	47	352	MUT
7427	2						1			2	3	5	133	MUT
7430					1		5			2	3	8	30	MUT
7432					8		3			10	3	21	133	MUT
7434			3				2			2	3	7	125	MUT
7436					3		2			1	3	6	39	MUT
7438							2	2		1	3	5	64	MUT
7440							1	5	4	3	4	13	174	—
7442							2	8		3	3	13	198	MUT
Total tumours	7	2	3	3	11	5	36	19	6	31				
Total inserts	29	3	6	5	179	11	129	80	23	210		675	5270	
Average per tumour												16	123	

The elements that transduce unique sequence are listed individually, while all inserts mapped to L1s or polyA in the genome are combined in column marked ‘?’, because it is not certain that they were copied from any specific L1. Elements active, number of different elements active in a given tumour. Inserts by discordant reads, total inserts found from discordant paired reads. PolyA, candidate inserts found by searching for tumour-specific polyA. TP53 mutation, mutations in TP53 called from Illumina sequencing. MUT, mutated; −, no mutation detected. † Note that tumours 3111 and 7404 had verified tumour-specific inserts of the chr12_A element, so definitely had some mobile-element activity

Combining the inserts found by discordant read pairs and our polyA search (Table 2), most of the tumours have evidence of somatic mobile element activity. Only 10/43 (23 %) tumours did not have either five or more inserts detected by discordant pairs or >50 candidate inserts detected by polyA (Table 2), and one of these (7404) had a verified insert (Additional file 2). On the other hand, there are a few tumours that may well not have activity, notably the 4/43 tumours that have zero or one unverified insert detected by discordant reads.

### Effects on genes

Among the inserts found by analysing discordant reads pairs, 198/675 (29 %) were in a gene listed by Ensembl. Of these, approximately 90 were in the same transcription direction as the gene, 108 in the opposite orientation. (These are approximate figures because only one junction was detected in most cases, and the orientation of this junction could be misleading in cases with 5’ inversion).

Five inserts seemed particularly likely to affect gene function: four were in coding sequence, of *MRPL13* and *ZAN* (both confirmed by PCR), *SYCP1* and *ZDHHC14*; and one was in the 3’ UTR of *PDE10A* (confirmed).

Several genes had inserts in more than one tumour (not confirmed by PCR): *AGBL4*, *DLG2* and *SNTG1* had insertions in three tumours; *AGBL1*, *AREG*, *EDIL3*, *EPHA6*, *GPM6A*, *LRP1B*, *NEGR1*, *NRXN3*, *PGCP*, and *PLAKHA4* had insertions in two tumours. *AGBL4*, *ROBO2*, *EYS* and *RYR3* appeared to have two insertions in distinct sites in the same tumour, though these could be insertions at a single rearrangement junction, since this is known to occur [28].

Among the insertions detected by polyA search (Additional file 3), which are less reliable and may be too small to affect gene function, a number of genes had inserts in several tumours, but these were very large genes (0.8 - 2.2 Mb) such as *LSAMP*, so the significance is unclear (Additional file 3).

## Discussion

### Mobile elements found

Our search for novel polyA sequence showed that retrotransposition occurs in the majority of oesophageal adenocarcinomas—40/43 had 10 or more candidate insertions (Table 2)—and the number of novel inserts, though very variable, was often in the hundreds according to the search for tumour-specific polyA runs.

Mobile element activity has previously been explored in several cancer types, by various methods. Some relied on L1 sequence to permit single-ended PCR of junctions or capture of junction fragments by hybridization [7, 9, 10]; others have used Illumina whole genome sequencing and bioinformatic tools such as TEA and TranspoSeq (which rely on presence of L1 sequence) [8, 14] and TraFiC (which also detects transductions that lack L1 sequence) [13] to identify likely L1 insertion and transduction [12] events; while Rodic *et al.* [29] used staining with well-validated polyclonal and monoclonal anti-LINE-1 ORF1p protein on tissue microarrays to detect ongoing L1 activity. Together these suggest that carcinomas have more L1 activity than non-epithelial cancers, with more than half of cases having detectable ORF1p protein and detectable inserts. Among carcinomas surveyed, lung NSCLC, head-and-neck squamous and colorectal had relatively more inserts identified than breast and prostate [7, 8, 10, 12–14] (though most breast cases stained for ORF1p protein [29]) . The high variability between cases of a carcinoma was illustrated by Lee *et al.* [8] who detected 2 to 15 inserts in 4 of 5 colorectal cancers but 106 in the remaining case, which also showed hypermutation and high CpG island methylation (CIMP-high).

Allowing for differences in approach, our results (Table 2) are consistent with these surveys, and probably place oesophageal adenocarcinomas among the tumour types with more frequent and abundant inserts, such as colorectal and lung cancers. TraFiC [13] detected 0 to 565 inserts in 36 lung cancers, with 4 having 187 or more, and 75 % of patients with at least some; while 15 colorectal cancers had 0–66 inserts, with only one tumour negative.

Our identification of candidate inserts simply by polyA has the advantage of high sensitivity but might be less robust than more stringent approaches. However, PCR across the candidate insert sites—which would fail for larger inserts and so underestimates our accuracy—succeeded in amplifying an insert with retrotransposon-like characteristics in about 85 % of cases, and manual curation of reads from 20 low-confidence examples showed that all had additional split reads that supported the presence of an insert. We might have expected occasional inserts to be polymorphisms overlooked in the matched normal, but all that amplified in our unbiased sample were unique to the tumour, only about 1 % were found among known polymorphisms [13, 25, 26], and only one or possibly 2 of 20 manually curated examples had any evidence they were germline.

The mobile element inserts we identified had the expected structure, allowing for known variations such as inversion of the 5’ end, as explored in more detail by others [8, 10, 13]. Among the less familiar variations, one of the inserts found in our polyA search, 7416_10, had polyA at both ends. Extreme examples of truncation were found, including one insert of polyA plus 1bp and target duplication (insert mapped to chr15 locus, inserted into chromosome 16; Additional file 2).

As expected, all the intact L1s implicated in formation of our inserts were from family L1HS, the human-specific L1 family, thought to be the only currently-active L1s. Most were in the original list of active ‘hot L1s’ prepared by Brouha *et al.* [24], based on December 2001 genome data plus functional assay of the L1’s activity *in vitro*. Our list includes their three most active L1s and eight of our list are in their top 20. However, this is biased by our use of the hot L1s to find examples. One of our original three active L1s identified purely from read pairs, on chromosome 20, was not listed by Brouha *et al.* [24], but matches their consensus sequence 99.6 %. Two of the most active elements, the Chr 3 and Chr 14 elements, are L1s absent from the reference but present respectively in 77 % and 25 % of cases examined by Tubio *et al.* [13].

We did not find positive evidence of Alu mobilisation. Although we found discordant reads aligned adjacent to Alus on chromosome 12 and 15, these alignments were essentially to their polyA tails, as discussed below.

### Why are the transduced sequences unique?

A puzzling feature of the 3’ transduction events is that the sequence transduced is unique in the reference genome. This is paradoxical since, if the mobile element were active in the germline, there should be multiple copies in the reference genome. It follows that there may be 3’ transduced sequences that are not unique in the genome because they *have* already been copied. Like L1s, new inserts of these sequences will not be mapped by aligners and so will usually go undetected.

### Activation of multiple elements

As also noted by others [13, 14], the 3’ transduction events, which identify the L1 that gave rise to the insert (Table 2) show that, where inserts were detected in a tumour, several elements were often mobile, rather than individual elements becoming active singly (Table 2). However, two of the three tumours with 12_A inserts had no other junctions detected, and the relative number of inserts of elements 22 and X_A varied from 6:0 in tumours 3129 and 3135, to 1:5 in tumour 7440, suggesting some differential activation, unless detection is skewed by technical details such as the aligner used, different fragment size and effective coverage in sequencing of the two tumour batches. Differences in activity of the chromosome 3 and 14 elements are of course partly determined by whether or not the individual has the element.

### Activation and genetic instability

An interesting corollary is that activation of these elements might constitute a new type of genetic instability in cancer. However, we cannot say whether L1s are specifically activated in these tumours or are also active in normal cells, because the non-cancer cells may be too polyclonal for new inserts to be detected. Also, even if activation is cancer-specific, it is not necessarily a distinct genetic instability phenotype: in particular, p53/*TP53* mutation may be permissive for cells to survive activation of retrotransposons [29, 30], and activation may be just one consequence of p53 loss. Almost all these Oesophageal adenocarcinomas are p53 mutant (Table 2; [22, 31]), and two (3119 and 3125) of the three tumours where no insertions were detected by discordant reads were two of the five tumours with no detected p53 mutation (Table 2).

### Do the inserts mutate genes?

Mobile element insertions can alter genes in various ways [1]. Apart from direct insertion into exons—as in the APC gene in a colon cancer [6]—they can terminate transcription by providing a polyA addition site, either the site used in creating the insert mRNA or, when in reverse orientation, an antisense polyA addition site about 0.5 kb from the 3’ end of the consensus L1 sequence [32]. But the 5’ UTR can also activate expression, in either orientation, since it includes a potent antisense promoter.

Some of our inserts might be in driver genes. The insert shown in Fig. 3 is in ENPP2, which is a nucleotide phosphodiesterase and phospholipase. It is fused in the breast cancer cell line ZR-75-30 [33] and may be upregulated in cancers. Of the genes listed in Results—with inserts in exons or almost in exons, or with multiple inserts—ROBO2 is a candidate tumour suppressor [34], and is a target of insertions in colon tumours [10].

Among the genes with single inserts in introns, detected by discordant read pairs, are several identified elsewhere as likely drivers: CNBD1 reaches driver status in exonic mutations [35], while RSPO2 is a known oncogene [36]. Tubio *et al.* [13] recorded an insert in CNBD1 also. CNTNAP5 was highlighted as relatively frequently mutated in exome sequencing of oesophageal adenocarcinoma [22, 31].

Activation of genes by inserts may be important. Davoli *et al.* [37] analysed reported patterns of mutation to identify likely tumour suppressors and oncogenes. We found many inserts in genes scored with moderate confidence (score 3 out of 4) as oncogenes—COL22A1, CSMD3, GaBRG2, PGM5, RALYL, RGS22, RSRC1, and TRPS1—while no inserts were in candidate tumour suppressors at confidence 3 or 4. Several of these genes and SYCP1, mentioned above, are involved in gene fusions: RGS22, SYCP1 and TRPS1 in breast cancer cell lines [33, 38], and RSPO2 in colon cancers [39]. This hints at insertional activation.

### How inserts are detected by paired-end analysis

We classified the inserts we found based on how they were identified by aligning sequences to the reference genome (Table 1). Most current strategies for analysis of structural variation in the genome by paired-end sequencing, as generated by the Illumina technology, rely on matching sequences to the reference genome, then looking for such abnormalities as ‘discordant read pairs’ where the reads map an abnormal distance apart, e.g. to different chromosomes; or ‘split reads’, where different parts of the same individual sequence read map to different locations in the genome [20].

Mobile element insertions give a variety of unexpected behaviours in such analysis. The first class of inserts we identified were inserts that include unique sequence located 3’ to L1s and had been transduced: these inserts appear to be junctions between the unique sequence and somewhere else in the genome. Many of the inserts appear to be translocations because the other end of the insert is repeat sequence, and therefore is not identified by discordant reads. We identified nine L1HS elements that transduce sequence this way, eight of which have been recorded by Tubio *et al.* [13], who described a total of 72. These inserts subdivide according to whether the L1 is in the reference genome or not (Table 1). Many recent L1 insertions in the germline are active, and are polymorphic and absent from the reference genome [13, 25, 26, 40]. Transduction by non-reference L1s is more difficult to identify because it requires a list of all active polymorphic L1s or a search of the matched normal genome for L1s. Our two examples—the chr 3 and chr 14 elements—were only detected by us because they are present in many individuals and very active, so produced large numbers of apparent breakpoints within a kilobase or so.

The second major class of inserts detected by discordant read pairs were inserts that the aligner software could map uniquely to a mobile element or its associated polyA, in the reference genome. These also resembled translocations to, or insertions of, the reference element. The inserts did not necessarily come from that element, because the alignments may have depended on the presence of polyA. Most striking were the chromosome 12 and chromosome 15 ‘elements’, which are the longest and third-longest polyA runs in the reference genome. The Novoalign aligner, which has enhanced ability to trim reads to obtain a match, confidently mapped reads here merely because they contained a long polyA string. In raw sequence alignments we also found reads aligned to the second-longest polyA run, 83bp at chr6:160521756–160521835, but only one junction with this mapping was unique to a tumour and so appeared as a candidate junction.

Finally, some apparent ‘translocation junctions’ were not even junctions, but occurred when read pairs within a mobile element insert were mapped to two distinct loci (Fig. 1E). Typically, one read of a pair was in transduced sequence, the other was in the polyA tail and mapped to one of the long polyA runs in the reference.

### Consequences for paired-end sequencing

Our data highlight difficulties and pitfalls in identifying mobile element insertions in paired-end sequencing data, and in distinguishing them from genomic rearrangements, some of which have already been flagged by Pitkänen *et al.* [12] and Tubio *et al.* [13].

Searches for mobile element inserts will have to allow for truncation and transduction, which result in inserts that may have little or no sequence from the mobile element proper [13, 18]. Of our 15 sequenced polyA inserts, all but one had target site duplications, as expected of non-LTR retrotransposons, but only 11/15 had L1 sequence in the insert, while the other 4 had unique sequence or a fragment that was unrelated to L1 and too small to map. TraFiC [13] attempts to address this problem.

It will also be difficult to achieve high sensitivity. To identify inserts from discordant reads, the reads have to fall in exactly the right places (Fig. 1), reducing effective coverage, and many inserts are too small to accommodate a whole read—of the 15 polyA inserts we sequenced, four were smaller than 100bp excluding the polyA, and one was only 13bp + 49 As. Split read analysis will help (Additional file 4), but may introduce additional artefacts such as ‘split mappings’ (Fig. 1E). It may be helpful to flag and isolate runs of polyA as a specific feature of reads.

Mobile element inserts create difficulties for interpreting structural rearrangements of the genome. Inserts maybe misidentified as translocations [12]. For some inserts, junctions will be found at both ends, but these are still indistinguishable from reciprocal translocations, especially since translocations may have duplication or deletion at the breakpoints [23]. Often there is no sign that the insert is an insert, because the junction 5’ relative to the mRNA intermediate is not unique and so not detected. Cataloguing transducable sequences will help, but since some somatically acquired L1 insertions can go on to generate new insertions [13], such catalogues will never be exhaustive. Again, searches for novel polyA may help.

Finally, our results illustrate that different aligners will produce different artefacts.

## Conclusions

New mobile element insertions are abundant in oesophageal adenocarcinomas. Since inserts may inactivate or activate genes, they are likely to contribute to mutation burden. The inserts create a variety of problems for the interpretation of paired-end sequencing as currently performed. Many element insertions will be missed in searches for L1s, because they are truncated and carry little or no L1 sequence. Conventional searches for structural rearrangements of the genome will miss inserts because their ends consist of repeat sequences and polyA, which cannot be mapped to the reference genome by aligner software. Even those that that are detected may resemble structural variants, and they may be difficult to identify with mobile element activity because the insert will not always contain retrotransposon sequence [18].

## Methods

### Tumours and cell lines

Batch P (numbered 3108–3323) were the 22 discovery cohort oesophageal adenocarcinomas with matched normal squamous epithelium or blood as described [22]. Batch B1 (7394–7442) were a further 21 tumours all with matched blood (Additional file 5). Briefly, the tumours reflected the known clinico-demographic features of the disease: male predominance (6:1), mean age 68 years (53 to 82), and mostly advanced disease (33/43 > stage I). The study was approved by the East of England-Cambridge South National Research and Ethics Service Committee (Research Ethics Committee Numbers 10/H0305/1 approved 17/02/2010 and 07/H0305/52 approved 28/08/2007), and all patients gave individual informed consent. Sample collection and DNA extraction were as described [22]. Authenticated Oesophageal adenocarcinoma cell lines [41] OE33, JH-EsoAd1 and FLO-1 were obtained from the originators or European Collection of Cell Cultures (ECACC) (OE33), and identity checked by short tandem repeat (STR) analysis.

### Paired-end sequencing

100bp paired-end sequencing was performed, on a single library per sample, under contract by Illumina on the Hi-Seq-2000 (Illumina, San Diego, CA) to a depth of at least 50x. Two protocols were used: Batch P and matched normals were sequenced to a mean coverage of 63-fold, using a PCR-amplified library of gel-purified fragments of median size 277-367bp, median absolute deviation 15 – 46. Some of these libraries contained large numbers of apparent small inversions, possibly caused by circularisation of fragments with single-stranded ends during library preparation, so inversions of less than 10kb were discarded. Occasional read pairs differed from another read pair by only one or two basepairs at one end, presumably PCR duplicates where a sequencing primer had lacked a terminal base [33, 42]. Batch B1 tumours were sequenced using TruSeq DNA PCR-Free protocol, giving median fragment sizes of 290–343, median absolute deviation 60 – 75. Few if any artefactual small inversions were present and no imperfect PCR duplicates. Sequencing of the cell lines was performed under contract by Beijing Genomics Institute similarly to batch P, with insert size around 500bp.

After quality control [22], read sequences were aligned to human reference genome GRCh37/hg19 using Burrows-Wheeler Alignment (BWA) [43], exact PCR duplicates removed using Picard [44]. For Batch B1 only, reads that did not map normally were re-aligned with Novoalign (Novocraft Technologies, Selangor, Malaysia). Rearrangements (structural variants) were identified from discordant read pairs, i.e. read pairs that failed to map to the reference genome at the expected separation and orientation [20], essentially as described [33, 45]. Discordant reads were sorted into clusters then separated into subclusters that supported the same rearrangement junction. For Batch P, we required rearrangements to be supported by at least three read pairs in the tumour, but without any read pairs in either the matched normal or any other normal from the same batch, i.e. 19 or 20 additional normals. For Batch B, with fewer known library artefacts, we also accepted rearrangements supported by only two reads. Around 10 % of rearrangement junctions were discarded because there was a plausible normal alignment of the pair of read clusters (90 % identity between mapped position and expected normal location, over at least 100 bp).

The polyA search identified sequence reads that contained 20 consecutive As or Ts and were paired with reads that had been confidently mapped by the BWA aligner (without Novoalign), mapping quality ≥30. Sequences were discarded if ≥80 consecutive bases had the lowest base quality score (marked ‘#’ in the BWA output), and inserts were discarded if there was polyA in the reference genome within 500bp of the mapped position (<10 % for most tumours).

### Verification

PCR primers (Additional file 2) were designed for annealing temperatures of 59 to 66°C. PCR products obtained from amplification of whole inserts were cloned and plasmids sequenced by Sanger sequencing. Products from individual junctions were sequenced directly. Where PCRs failed new primers were designed. Putative inserts discovered by discordant read pairs were sampled for verification first from tumour 3320, then choosing examples from tumours for which we already had DNA readily available. Inserts with reads at both ends were preferentially sampled. To sample polyA inserts for verification in an unbiased way, an insert was chosen that was nearest to each 100 Mb interval along the genome, taking different tumours, two of high and two of low cellularity, as judged by the size of copy number steps. This sampling preceded the comparison with known polymorphisms.

### Ethics

The study was approved by the East of England-Cambridge South National Research and Ethics Service Committee (Research Ethics Committee Numbers 10/H0305/1 approved 17/02/2010 and 07/H0305/52 approved 28/08/2007) and all patients gave individual informed consent.

### Availability of data

The sequence data are available in bam format at the European Genome-phenome Archive (EGA), in accordance with International Cancer Genome Consortium (ICGC) practice, Accession number EGAD00001001048.

## Additional files

Additional file 1:
**Table of apparent insert junctions from discordant read pair analysis that map to the clusters of breakpoints in Table** 1 **.**
Additional file 2:
**Table of sequenced mobile element inserts or insert junctions, amplified by PCR, with primers.**
Additional file 3:
**Table of candidate inserts detected by searching for polyA.**
Additional file 4:
**Examples of inserts scrutinized on the Integrative genomics viewer (IGV).**
Additional file 5:
**Tumours, demographic data.**
Additional file 6:
**Participants in the OCCAMS consortium and associated sequencing project.**

## Abbreviations

ICGC: International Cancer Genome Consortium
L1: Long interspersed element 1
LINE: Long interspersed element
LTR: Long terminal repeat
OCCAMS consortium: Oesophageal Cancer Clinical And Molecular Stratification consortium
PCR: Polymerase chain reaction
SINE: Short interspersed element
SVA element: Composite SINE-R, VNTR, and Alu element
UTR: Untranslated region
