# Supplementary material for: Mobile element insertions are frequent in oesophageal adenocarcinomas and can mislead paired-end sequencing analysis
Source: BMC Genomics. 2015 Jul 10;16(1):473. doi: 10.1186/s12864-015-1685-z (PMC4498532; doi:10.1186/s12864-015-1685-z)

Additional file 4. Examples of inserts scrutinized on the Integrative genomics viewer (IGV)

A sample of candidate inserts that had been found by searching for polyA were scrutinized manually to see whether they resembled tumour-specific, L1-mediated insertion events.

Candidate inserts were selected for scrutiny from tumour 7396 that had the highest number of candidate inserts, approximately 850, and its matched normal 7395. We sampled the *least-confident* inserts—those supported by only 3 reads—and examined every 7th insert of 140 to give a sample of 20, using the Integrative Genomics Viewer (IGV), illustrated by Screenshots 1 to 3 below.

Data is summarized in the table. It suggests that these are all, or almost all, L1-related inserts; and that only 1/20 sampled is not tumour-specific and hence a germline polymorphism. All candidate inserts were flanked by discordant read pairs additional to those identified by our search for polyA. All also show split ('soft-clipped') reads that support a specific point of insertion, in all but one case 4 or more, and with one striking exception—clearly a polymorphism (Screenshot 3)—these are absent from the matched normal. The sequences and starting points of the unmatched part of the split read sequences argue that the inserts are L1-mediated events: the sequences are either polyA/T, or match L1 sequence or, in one case seem likely to be transduced sequence. Usually both polyA and L1 sequence are present often with a gap between consistent with target site duplication (Screenshot 3). The only example *not* showing identifiable L1 or likely transduced sequence is the polymorphism, but it still shows target site duplication (Screenshot 3). In addition to the split reads, many discordant reads mapped near the insert sites in the tumour. The paired sequences are unmapped or map all over the genome, and at least some map to L1 sequence (the BWA aligner typically maps to a random example of L1 and reports zero mapping quality to indicate there are other matches). The few discordant reads in the normals (though not the tumour) were scrutinized to see whether they could support the insert, particularly whether they flanked the insert site in the correct orientation. Other than the clear polymorphism (Screenshot 3), only one other insertion, ID 55562, has a read pair in the normal that could support the tumour insert: the unmapped paired read is largely A. However, the read maps across the tumour insert site and the tumour has 16 split reads, while the normal has none, arguing against a germline event (Screenshot 2).

Table. Features of reads around insert sites from IGV, Tumour 7396.

Cluster ID	Insert site	Reads found in normal		Reads found in tumour			
		discordant reads	split reads	discordant reads	Split reads, not polyA		split reads polyA/T
					number	sequence	
3755	chr1: 191,875,449	0	0	13	6	L1	1
6947	chr2:79,342,454	0	0	5	5	L1	3
11605	chr3:62,275,353	0	0	12	1	L1	3
13395	chr3:156,908,677	1(W)	0	16	8	L1	0
16752	chr4:117,448,272	2(N+N)	0	17	3	L1	5
20124	chr5:90,928,732	0	0	15	5	L1	5
21646	chr5:164,825,226	0	0	13	8	L1	2
24576	chr6:117,123,510	1 (C)	0	11	5	L1	2
27487	chr7:78993840	1*	0	12	6	transduced?	9
28737	chr7:133,118,167	0	0	4	1	L1	0
30656	chr8:65,772,213	N	0	16	5	L1	9
32816	chr9:17,034,145	0	0	13	2	L1	3
36140	chr10:57,114,774	2(W + N)	0	5	2	L1	5
38164	chr11:21,359,408	2(W+W)	0	37	15	L1	10
41185	chr12:38,694,701	3 (W+C+C)**	0	6	4	L1	2
44217	chr13: 62,745,208	0	0	28	14	L1	5
46878	chr14:82,593,554	0	0	3	3	L1	2
50149	chr16:51773,356	N	0	10	1	L1	6
55562	chr19:31,965,822	3(N + W + 1)***	0	18	5	L1	11
59543	chrX:20985127	1	19+13, polyA in both directions	3	0	N/A	28+15, polyA in both directions

Cluster ID: arbitrary ID generated during search, before filtering. Insert site: likely insert site identified as split point of split ('soft-clipped') reads; this may not be exact because of target site duplication or deletion. Reads found in tumour and normal: reads within a window of approximately 600 bp spanning the likely insert site that are not normal. Normal discordant: discordant read pairs in matched normal with pairs unmapped or mapped to another chromosome, given as number of discordant reads in the interval, with details in brackets. These discordant reads were individually examined for compatibility with the insert position defined in the tumour: N indicates that the paired read was mostly Ns or did not have a mapping using BLAT; W, a read with wrong orientation, i.e. incompatible with supporting an insert; C a read

that crosses the insert site found in the tumour and therefore is unlikely to support the insert. Normal split: split reads in normal. Tumour discordant reads: total discordant reads in the interval. Tumour Split, not polyA: split reads whose sequence is not polyA or T and show a consistent breakpoint and unmatched sequence. Sequence: nature of the unmatched sequence. One, marked ‘transduced?’ maps to chr3: 181972623-181972692 which itself is a complex candidate insert cluster 14005M identified by the polyA search—this seems likely to represent sequence transduced from the chr3 locus. Tumour split polyA or T: number of split read sequences whose unmatched part is polyA or T, with consistent start point.
* Paired read maps to L1 e.g. at chr1:42988221 (+), but breakpoint in tumour is in the middle of this read
** These three read pairs not only do not match any of the tumour read pairs but do not match each other—the paired sequences map to unique sequence at three unrelated loci chr3:107424583 (+), chrX:26670976 (+), and chr2:137616613 (-)
***One of three read pairs might support insertion in the normal; its pair is TTGAGTGTGCTAAATTAAAAATAAAAAAAACC AAAAAAAAAAAAAACAAAAAAAAAAAAAAAACATCTAAATTTAATAAAATAAAAAAAAAAAAAAAAACA

Screenshots 1 to 3, showing reads on the Integrative Genomics Viewer, IGV.
Horizontal scale is genome, with chromosome position shown at top. At the bottom a short blue bar marks the position of the cluster of reads identified, spanning the ends of the component reads’ ends proximal to the As or Ts. In the middle two panels, representing the normal (above) and tumour (below), grey horizontal bars are individual sequence reads that are paired in a normal read pair. Grey boxes with red outline, reads that are paired with reads that could not be aligned - in many cases these are the reads we identified as containing polyA. Other uniformly coloured bars are reads whose paired were mapped to other chromosomes, at least in some cases to an arbitrary L1 sequence. Partially-coloured reads in green, red or multicoloured are ‘split’ reads that align for part of their length and then deviate, the colours representing A, T, C or G, so polyA and T are green and red respectively. These define the insertion site and show the sequence of the insert joined at each junction.

Screenshot 1 shows a typical insert; Screenshot 2 is an example that might be considered uncertain, because there is one read in the normal that might support the insert being germline, though unlikely; Screenshot 3 shows the one out of twenty that clearly was a polymorphism. It appears to represent germline insertion of sequence with polyA at both ends, with a short target site duplication.

Screenshot 1: Typical insert, at chr5:90928545, cluster ID 20124. For explanation see text above.

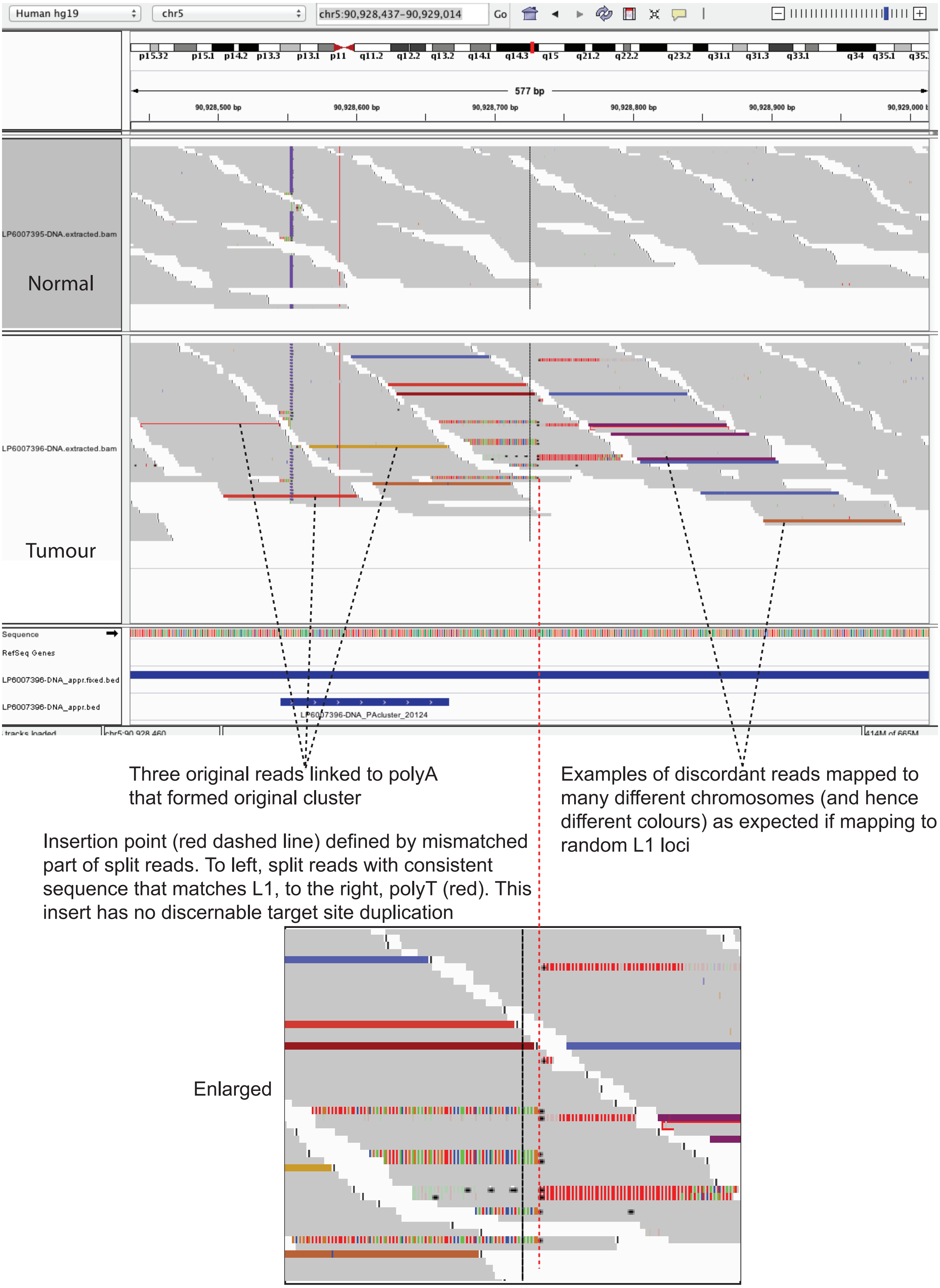

Screenshot 2: Insert at chr19:31,965,822, cluster ID 55562. Example with 3 discordant reads in normal, labelled X,Y,Z. X and Y are in wrong orientation, mapped reads point away from the insert, and X's pair is unmapped because it is mostly Ns. Read Z is paired to largely PolyA sequence (see above), but it maps perfectly without clipping chr19:31,965,800-31,965,899* - across the insert site which is at 31,965,822. There are 16 split reads in the tumour, none in the normal. It seems very unlikely this is a germline insertion.

*AAAATCTATAAATCTTTTTCTATAAGATTTATTCTACTAAGGTTTTGTAA
CAACAGAGAAAAATGTCTATGTTATAATTTTAGGTTAAAAGACAAGGTAC

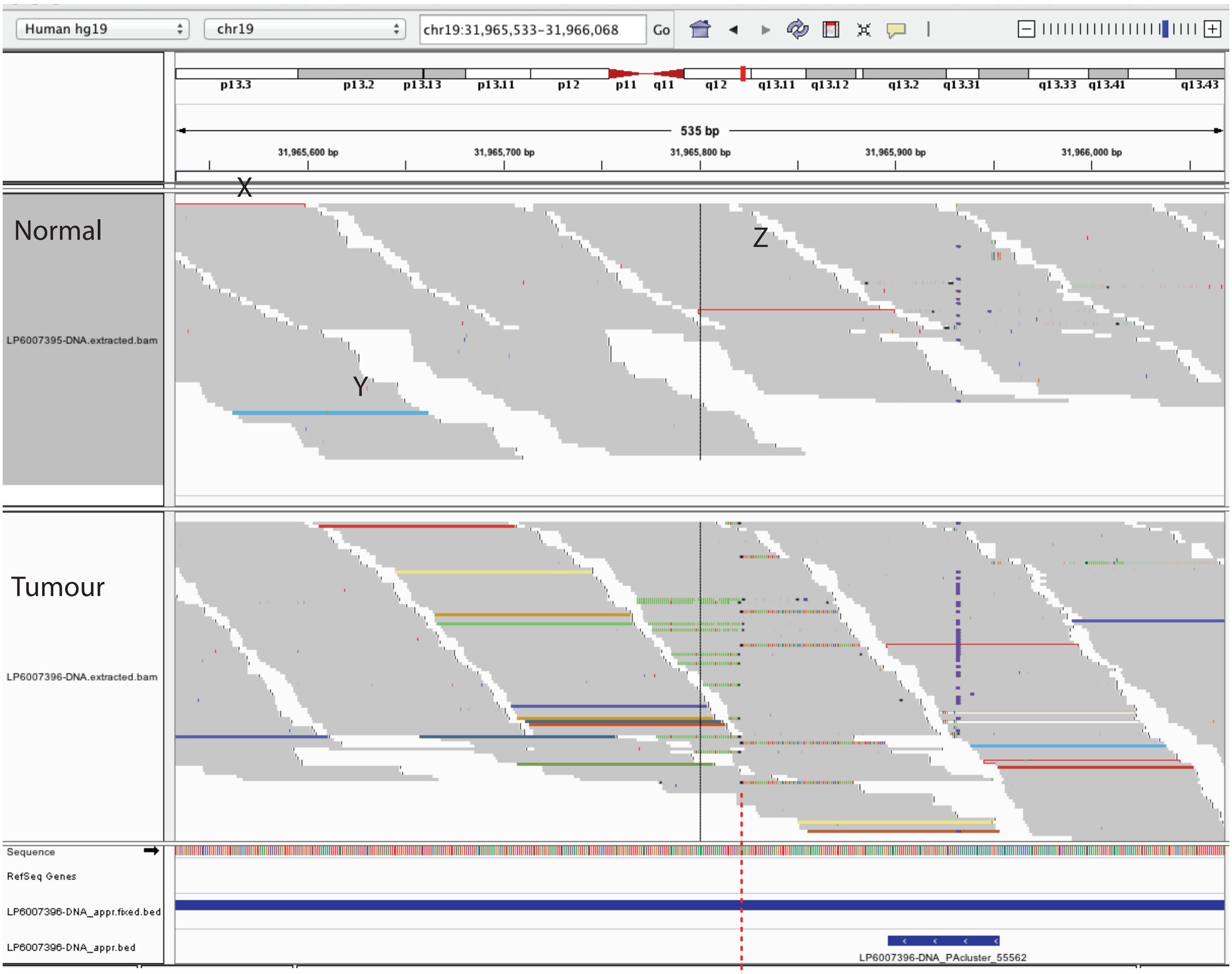

Insertion point (red dashed line) defined by mismatched part of split reads. To right, split reads with consistent sequence that matches L1, to the left, polyA (green). This insert has no discernable target site duplication

Enlargement of split reads

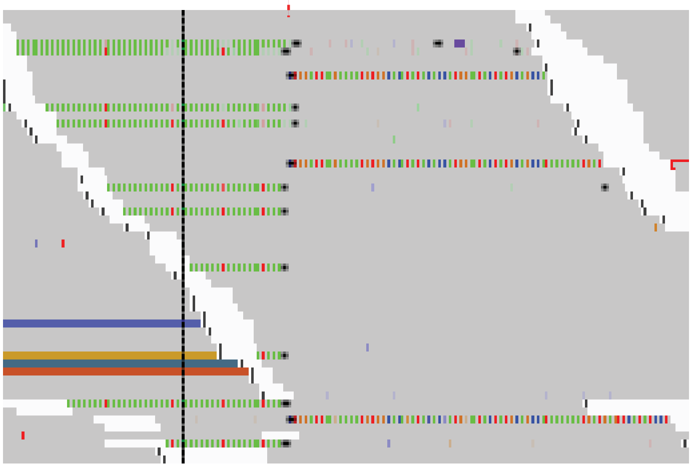

Screenshot 3: Germline insert at chrX:20985127, cluster ID 59543. In this one case that is a polymorphism, there are multiple split reads (green) in the normal.

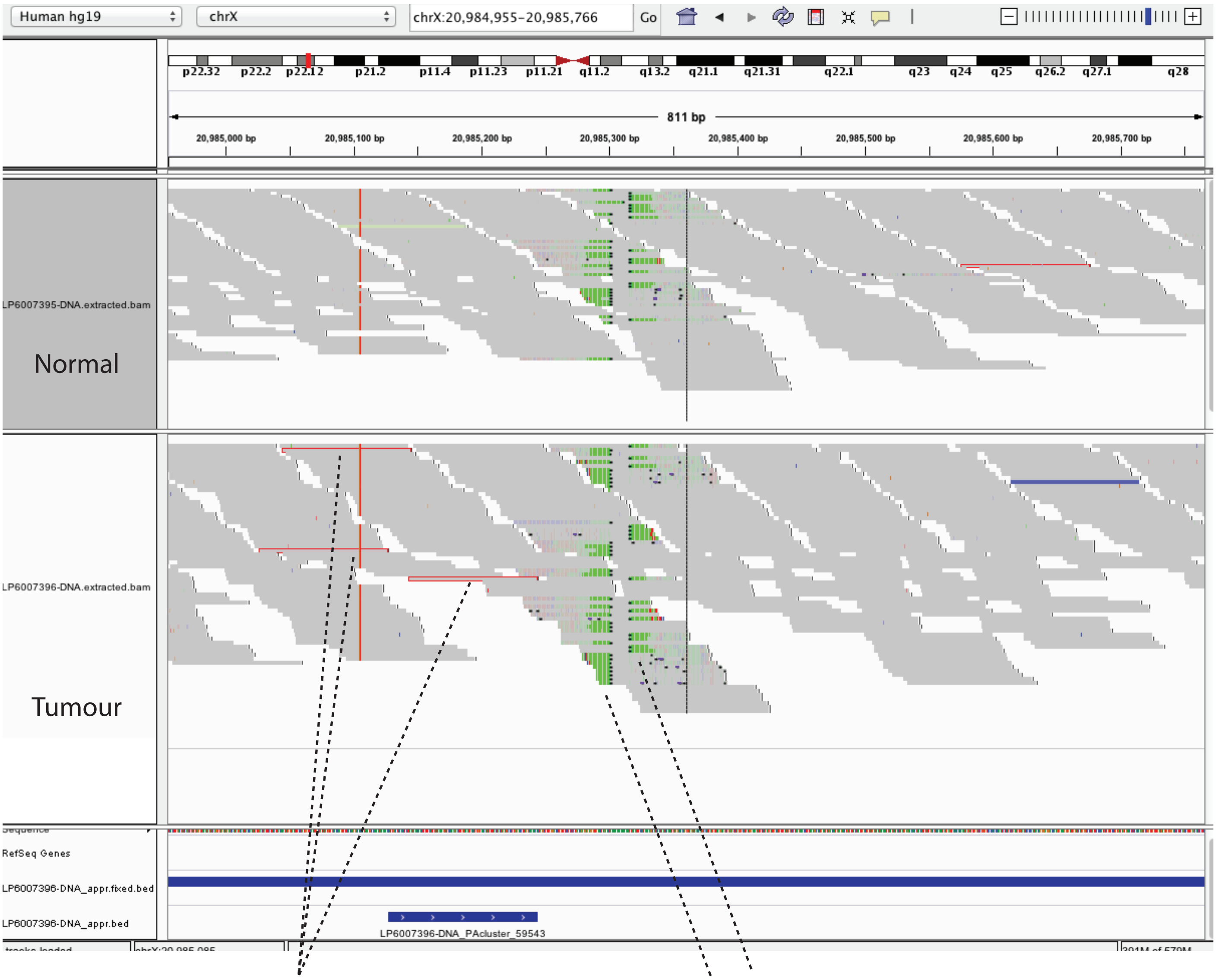

Three original reads linked to polyA

Multiple split reads, in both orientations, whose mismatched end is polyA (green). Note gap between ends of split sequences, consistent with target site duplication

Enlargement of split reads

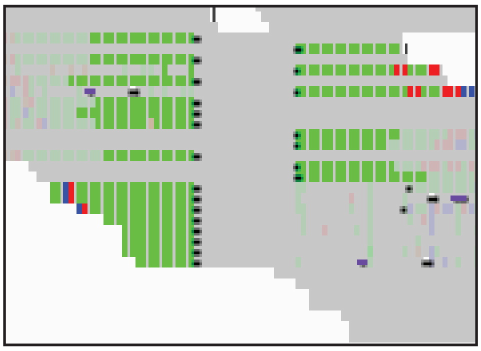

Supplement: Additional file 4: — Examples of inserts scrutinized on the Integrative genomics viewer (IGV). [file 12864_2015_1685_MOESM4_ESM.pdf]
