# Supplementary material for: Mobile element insertions are frequent in oesophageal adenocarcinomas and can mislead paired-end sequencing analysis
Source: BMC Genomics. 2015 Jul 10;16(1):473. doi: 10.1186/s12864-015-1685-z (PMC4498532; doi:10.1186/s12864-015-1685-z)
Supplement: Additional file 5: — Tumours, demographic data. [file 12864_2015_1685_MOESM5_ESM.pdf]

Additional File 5: Demographic data of Tumours Batch B1

Case ID	Gender	Age (yrs)	Chemo treated	Differentiation	T	N	M	Stage	Alive/ Deceased	Survival (months)	Normal sequenced
7394	Male	63	Yes	moderate	3	1	0	III A	Alive 10/2013	At least 31	Blood
7396	Female	69	Yes	poor	3	1	0	III A	Deceased	38	Blood
7398	Male	76	Yes	poor	3	2	0	III B	Deceased	14	Blood
7401	Male	74	No	Mod/well	1b	0	0	I A	Alive 05/2014	At least 36	Blood
7404	Male	67	No	poor	1	0	0	I B	Alive 01/2014	At least 33	Blood
7407	Male	74	Yes	moderate	3	1	0	III A	Deceased	13	Blood
7409	Male	65	Yes	moderate	1	0	0	I A	Deceased	13	Blood
7414	Male	53	No	moderate	3	2	1	IV	Deceased	10	Blood
7416	Male	69	No	NR	3	1	1	IV	Deceased	17	Blood
7418	Male	66	Yes	moderate	3	1	0	III A	Alive 08/2013	At least 22	Blood
7420	Female	79	No	moderate	3	3	0	III C	Deceased	23	Blood
7422	Male	71	No	moderate	3	0	0	II B	Alive 05/2014	At least 30	Blood
7424	Male	77	No	moderate/poor	3	2	0	III C	Deceased	9	Blood
7427	Male	66	No	poor	2	2	1	IV	Deceased	7	Blood
7430	Male	76	No	Moderate/poor	3	1	0	III A	Deceased	19	Blood PBMC
7432	Male	54	Yes	Moderate	3	2	1	IV	Deceased	12	Blood PBMC
7434	Male	57	Yes	Moderate	3	3	0	III C	Deceased	24	Blood PBMC

									Alive		Blood PBMC
7436	Male	73	No	Moderate	2	0	0	1B	22/05/2014	At least 37	
7438	Male	78	No	Moderate	2	1	0	II B	Deceased	2	Blood PBMC
7440	Male	72	Yes	Poor	2	0	0	I B	NR	NR	Blood
7442	Male	74	No	moderate	2	0	0	I B	NR	NR	Blood
