# Supplementary material for: Mobile element insertions are frequent in oesophageal adenocarcinomas and can mislead paired-end sequencing analysis
Source: BMC Genomics. 2015 Jul 10;16(1):473. doi: 10.1186/s12864-015-1685-z (PMC4498532; doi:10.1186/s12864-015-1685-z)
Supplement: Additional file 6: — Participants in the OCCAMS consortium and associated sequencing project. [file 12864_2015_1685_MOESM6_ESM.pdf]

Additional File 6: Participants in the OCCAMS (Oesophageal Cancer Clinical and Molecular Stratification) Consortium and associated Oesophageal Adenocarcinoma sequencing project, by affiliation

Medical Research Council (MRC) Cancer Unit, University of Cambridge, Cambridge, UK.

Jamie M J Weaver, Caryn S Ross-Innes, Mariagnese Barbera, Chin-Ann J Ong, Pierre Lao-Sirieix, Laura Smith, Nicola Grehan, Rebecca C Fitzgerald, Barbara Nutzinger, Zarah Abdullahi, Irene Debiram-Beecham, Jason Crawte, Shona MacRae, Ayesha Noorani, Rachael Fels Elliott, Xiaodun Li, Jan Bornschein, Sebastian Zeki, Hamza Chettouh, Nadeera de Silva, Eleanor Gregson & Tsun-Po Yang

Cancer Research UK Cambridge Institute, University of Cambridge, Cambridge, UK.

Nicholas Shannon, Andy G Lynch, Mark J Dunning, Mike L Smith, Charlotte L Anderson, Benilton Carvalho, Carlos Caldas, Matthew D Eldridge, Simon Tavaré, Lawrence Bower, Achilleas Achilleos & Maria Secrier

Department of Histopathology, Cambridge University Hospitals NHS Foundation Trust, Cambridge, UK.

Anna Paterson, Maria O'Donovan, Alison Cluroe, Ahmad Miremadi, Betania Mahler-Araujo & Shalini Malhotra

Department of Pathology, University of Cambridge, Cambridge, UK.

Paul A W Edwards

Oesophago-Gastric Unit, Cambridge University Hospitals NHS Trust, UK.

Richard Hardwick

Oxford NIHR BRC Informatics Programme, Department of Computer Science, University of Oxford, Oxford, UK.

Jim Davies, Charles Crichton

Department of Computer Science, University of Oxford, Oxford, UK.

Christian Schusterreiter

Salford Royal National Health Service (NHS) Foundation Trust, Salford, UK.

Stephen J Hayes, Yeng Ang, Anne-Marie Lydon & Soney Dharmaprasad

Faculty of Medical and Human Sciences, University of Manchester, Manchester, UK.

Stephen J Hayes

Wigan and Leigh NHS Foundation Trust, Manchester, UK.

Sandra Greer

Royal Surrey County Hospital NHS Foundation Trust, Guildford, UK.

Shaun Preston, Izhar Bagwan & Sarah Oakes

Edinburgh Cancer Research Centre, Edinburgh University, Edinburgh, UK.

J Robert O'Neill

Edinburgh Royal Infirmary, Edinburgh, UK.

J Robert O'Neill, Vicki Save & Richard Skipworth, Kasia Adamczuk

University Hospitals Birmingham NHS Foundation Trust, Birmingham, UK.

Olga Tucker, Derek Alderson & Philippe Taniere

Cancer Sciences Division, University of Southampton & University Hospital Southampton NHS Foundation Trust, UK

Timothy J Underwood, Jamie Kelly, James Byrne, Donna Sharland, Annette Hayden, Jack Owsley, Lisa Boulter, Fergus Noble & Bernard Stacey

Gloucester Royal Hospital, Gloucester, UK.

Hugh Barr, Neil Shepherd, L Max Almond & Oliver Old

St Thomas's Hospital, London, UK.

Jesper Lagergren, James Gossage, Andrew Davies, Robert Mason, Fuju Chang, Janine Zylstra & Christopher Peters

King's College London, London, UK.

Jesper Lagergren, James Gossage, Andrew Davies, Robert Mason, Fuju Chang & Janine Zylstra

Karolinska Institutet, Stockholm, Sweden.

Jesper Lagergren,

Plymouth Hospitals NHS Trust, Plymouth, UK.

Grant Sanders, Tim Wheatley, Richard Berrisford, Tim Bracey, Catherine Harden & David Bunting

Norfolk and Norwich University Hospital NHS Foundation Trust, Norwich, UK.

Tom Roques, Jenny Nobes, Suat Loo, Mike Lewis, Ed Cheong & Oliver Priest

Norfolk and Waveney Cellular Pathology Network, Norwich, UK.

Laszlo Igali

Nottingham University Hospitals NHS Trust, Nottingham, UK.

Simon L Parsons, Irshad Soomro, Philip Kaye, John Saunders, Vincent Pang, Neil T Welch, James A Catton, John P Duffy & Krish Ragunath

University College London, London, UK.

Laurence Lovat, Rehan Haidry, Haroon Miah, Sarah Kerr, Victor Eneh & Rommel Butawan

Cambridge University Hospitals NHS Foundation Trust, Cambridge, UK.

Hugo Ford, David Gilligan, Peter Safranek, Andrew Hindmarsh, Vijayendran Sudjendran, Andrew Metz Nicholas Carroll & Edmund Godfrey

Department of Pathology, University Hospital of South Manchester NHS Foundation Trust, UK.

Michael Scott

Department of Pathology, Peterborough and Stamford Hospitals NHS Trust, UK

Suzi Lishman

Department of Pathology, University Hospitals Coventry & Warwickshire NHS Trust, UK

Sari Suortamo

Supporting the OCCAMS consortium

The ECMC Network in Southampton, Edinburgh and Birmingham

The Queen Elizabeth Hospitals Birmingham Charities

The Human Research Tissue Bank, supported by the NIHR Cambridge Biomedical Research Centre
